# Biomarkers of lesion severity in a rodent model of nonarteritic anterior ischemic optic neuropathy (rNAION)

**DOI:** 10.1371/journal.pone.0243186

**Published:** 2021-03-25

**Authors:** Yan Guo, Zara Mehrabian, Mary A. Johnson, Neil R. Miller, Amanda D. Henderson, John Hamlyn, Steven L. Bernstein

**Affiliations:** 1 Department of Ophthalmology and Visual Sciences, University of Maryland at Baltimore (UMB), Baltimore, Maryland, United States of America; 2 JHU Wilmer: Division of Neuro-Ophthalmology, Wilmer Eye Institute, Johns Hopkins University School of Medicine, Baltimore, Maryland, United States of America; 3 Department of Physiology, UMB, Baltimore, Maryland, United States of America; 4 Department of Anatomy and Neurobiology, UMB, Baltimore, Maryland, United States of America; Bascom Palmer Eye Institute, UNITED STATES

## Abstract

The rodent model of nonarteritic anterior ischemic optic neuropathy (rNAION) is similar in many of its pathophysiological responses to clinical NAION. Like human NAION, there is significant variability in the severity of the lesion produced, and little is known of the parameters associated with rNAION induction severity or if pre- or early post-induction biomarkers can be identified that enable prediction of lesion severity and ultimate loss of retinal ganglion cells (RGCs). Adult male Sprague-Dawley outbred rats were evaluated for various parameters including physiological characteristics (heart rate, respiratory rate, temperature, hematocrit [Hct]), optic nerve head (ONH) appearance, pre- and post-induction mean diameter, and intravenous fluorescein and indocyanine green angiographic patterns of vascular leakage at 5 hours post-induction, performed using a spectral domain-optical coherence tomography (SD-OCT) instrument. Early changes were correlated with ultimate RGC loss by Brn3a (+) immunohistology. RGC loss also was correlated with the relative level of laser exposure. The severity of ONH edema 2d, but not 5hr, post induction was most closely associated with the degree of RGC loss, revealing a threshold effect, and consistent with a compartment syndrome where a minimum level of capillary compression within a tight space is responsible for damage. RGC loss increased dramatically as the degree of laser exposure increased. Neither physiological parameters nor the degree of capillary leakage 5hr post induction were informative as to the ultimate degree of RGC loss. Similar to human NAION, the rNAION model exhibits marked variability in lesion severity. Unlike clinical NAION, pre-induction ONH diameter likely does not contribute to ultimate lesion severity; however, cross-sectional ONH edema can be used as a biomarker 2d post-induction to determine randomization of subjects prior to inclusion in specific neuroprotection or neuroregeneration studies.

## Introduction

Nonarteritic anterior ischemic optic neuropathy (NAION) is the most common cause of sudden optic nerve-related vision loss in individuals over the age of 50 in the developed world [1]. In this condition, vascular decompensation occurring in the tightly restricted space of the optic nerve head (ONH) results in a compartment syndrome and sudden ischemia in the anterior portion of the optic nerve (ON). Human NAION is distinguished by ONH capillary incompetence, demonstrable by both intravenous fluorescein angiography (IVFA) and indocyanine green (ICG) leakage in early stages of the disease [2]. Direct tissue analysis of this disease is difficult to obtain, as NAION is a nonfatal disease. Clinical diagnosis instead relies on symptoms, signs, and electrophysiological findings (eg, visual evoked potentials (VEP) [3, 4].

To circumvent the problems inherent in NAION clinical research, we developed rodent and primate nonarteritic anterior ischemic optic neuropathy (rNAION and pNAION, respectively) [5–7]. These models are pathophysiologically similar in many ways to the clinical disease in terms of presentation with ONH edema, acellular and cellular inflammatory responses, ON-axon loss, isolated retinal ganglion cell (RGC) loss and visual debilitation [8]. In these models, laser-induced superoxide radicals from a photosensitive dye induce anterior ON capillary decompensation and leakage, ultimately resulting in ON edema, a compartment syndrome, and a lesion resembling the clinical one [9]. We previously documented fluorescein leakage in rNAION [6].

Despite extensive research using murine models of NAION [5, 10, 11], many questions remain concerning rNAION induction consistency, predictability of lesion severity, induction parameters, and if different NAION biomarkers, such as post-induction ON edema or capillary leakage, correlate with ultimate RGC and vision loss [12]. This problem is homologous to the variability in clinical lesion severity seen in NAION as well as in other central nervous system (CNS) ischemia/stroke models [13, 14]. This variability results in the statistical requirement to analyze large numbers of test animals to determine efficacy of proposed neuroprotective agents, thus requiring considerable time, effort, and cost. Identification of relevant early NAION biomarkers might improve selection of equivalently affected test animals prior to treatment randomization, potentially improving predictive accuracy of neuroprotective treatments, while using fewer animals.

In an attempt to improve lesion consistency in our rNAION model, we evaluated multiple variables potentially associated with lesion severity in an outbred strain with attention paid to the response variations among animals. Variables included correlation of pre-induction rodent ONH diameter with post-induction edema, because the risk of clinical NAION is associated with a small ONH (the ‘disk at risk’), as well as the intrinsic variables of heart rate and body temperature at induction. Additionally, we evaluated physical parameters of the ONH early (5hr) and late (2d) post-induction as potential biomarkers of model severity. These included: 1) ONH appearance and degree of ONH edema by direct visualization and spectral domain-optical coherence tomography (SD-OCT) and 2) vascular decompensation and capillary leakage. The purpose of this study was to determine the observable factors most closely associated with RGC death in rNAION in order to identify biomarkers of lesion severity.

As the rNAION lesion largely results from a compartment syndrome following vascular decompensation and leakage [4, 15], we reasoned that evaluation of early vascular leakage signal intensity could be used as a potential biomarker of lesion severity. We chose a time limit of 5 hours post induction because we have previously demonstrated that a neuroprotective rNAION treatment (PGJ_2_) started at this time point was effective [15].

## Methods

### Animals

All animal protocols were approved by the UMB institutional animal care and use committee. Sixty-nine male Sprague-Dawley and six male Wistar rats (200-250g) were used in this study.

### Anesthesia and rNAION induction

Animals were anesthetized with an intraperitoneal mixture of ketamine and xylazine (80mg; 4mg/kg, respectively) and kept on warming pads during anesthesia induction, rNAION induction, and recovery, to minimize core body temperature changes. The pupils were dilated with topical 1% cyclopentolate-2.5% phenylephrine, and corneas were topically anesthetized with 0.5% proparacaine. For animals analyzed 3–5 hr post induction, anesthesia was re-induced using a ketamine-xylazine mixture of 80mg/1mg/kg. Xylazine’s systemic effects, including bradycardia, hypotension, and respiratory depression [16], were reversed using atipamezole (1mg/kg IM). A rat fundus contact lens designed by us and now marketed commercially (Micro-R; Nissel and Cantor, UK) was placed on one eye using methylcellulose eyedrops. The retina and ONH were imaged using a Haag-Streit model 900 Goldmann slit-lamp biomicroscope (Haag-Streit; Bern, Switzerland). rNAION was induced using an intravenous injection of 1ml/kg of a 2.5mM solution of rose bengal in phosphate-buffered saline (PBS, pH 7.4) administered via tail vein. Thirty seconds post injection, the ONH was illuminated for 6–11 seconds with a clinical 532nm wavelength laser (Iridex Oculight GLx), using a spot size of 500μm diameter, 50mW power. Importantly, because different instruments can have slightly different outputs or change over time, we measured laser power output at the time of treatment using a laser power meter with a pyroelectric detector optimized for the appropriate wavelength and short duration (1 sec pulse) (Field Mate, Power Max, Coherent, Santa Clara, CA). Laser output varied from 43.5 to 48.1mW at the meter, depending on small variations in angle. Animals were maintained isothermally (38°C) using a circulating heating pad or by deltaphase pad and were removed from the pad only after recovery from anesthesia to prevent hypothermia that might alter their response to ON ischemia induction. Animals were allowed to recover and used for individual analyses. We have found that minimizing the time from rose bengal administration to laser induction is critical for maximizing lesion severity; even as little as a 30-second delay can cause significant changes in the relative lesion severity after induction. However, some interval must exist from dye bolus administration until dye equilibration within the circulation. We used a 30-second ‘mixing time’ from end of dye administration to start of laser induction. rNAION was induced in only one eye of each animal; the other eye was used as an internal (naïve) control. Animals were eliminated from study if there were delays in induction following dye administration.

### Systemic biomarker analysis

Thirteen animals were used in this part of the study. Animals were weighed before induction, and rose bengal was administered according to body weight at 2.5mg/ml/kg. Blood volume in rats is linearly associated with body weight up to 400g [17]. Hct was quantified using capillary centrifugation. Temperature was measured rectally, and heart rate immediately prior to induction was determined directly using a pediatric stethoscope, with counts obtained every 15 seconds X 4 to yield beats per minute (BPM).

### Local biomarker analysis

#### Fundus color photography

A Nikon D-40 digital camera with slit-lamp adapter was used for color fundus photos. Leakage analysis used two different dyes (Fluorescein and indocyanine green (ICG) because of their different properties. Because fluorescein is excited by visible light (peak 494nm), evaluation of deep/pigmented structures is limited. Additionally fluorescein binds poorly to serum proteins [18], diffusing away from damage sites. In contrast, ICG is excited by infrared light (peak ~780nm), enabling deeper tissue penetration and imaging through pigmented tissue [19]. ICG also is ~98% bound to serum lipoproteins [20], slowing diffusion through tissues and enabling identification of focal subzones of leakage. We reasoned that the intensity and/or accumulation of fluorescent signal would be an indication of the relative lesion severity, which then could be confirmed by later RGC stereology. Intravenous fluorescein angiography (IVFA) and intravenous indocyanine green angiography (ICGA) can be performed using different channels on the Heidelberg SD-OCT instrument, enabling nearly simultaneous FA and ICG pattern comparisons in the same eyes. Animals were induced for 6, 9, 10 or 11 seconds for these experiments.

Five hours post-induction, animals were re-anesthetized and separately intravenously injected with 0.07ml of a 10% solution of sodium fluorescein (fluorescite; Alcon, Geneva, Switzerland) and ICG. ICG concentrations were evaluated from 0.3 to 2% for optimal leakage signal; we ultimately used 0.1ml of a 5mg/ml (0.5%) solution of freshly prepared ICG in sterile dH_2_O for a 250g rat. This corresponds to 2mg/kg body weight. Both injections were completed independently within 30 seconds of each other, as ICG precipitates in saline, and co-administration with fluorescein depletes the ICG signal. We imaged the rodent retina *en face* through the same contact lens used for rNAION induction, coupled to the SD-OCT 25-diopter adapter lens. We initially switched between the two fluorescent-dye detection settings on the Heidelberg instrument. Optimizing individual instrument settings for the analysis was critical, as the Heidelberg instrument is capable of varying laser (excitation) power, detector sensitivity, and signal normalization, all of which will alter final signal output. We used both the 30- and 15-degree field examinations. For IVFA, power was 100 and the detector sensitivity initially was set to 50, without normalization. We used a laser output of 50–100% for both dyes and ICG detector set to 50, without normalization. ONH fluorescence was analyzed at 1, 3, 6 and 12 minutes post-injection. Optimized ICG final settings used 6 min, post-ICG injection with a 15-degree field and 50% power.

We attempted to evaluate further rNAION-associated deep focal ONH leakage using OCTA, which generates sequential images, each focused at progressively deeper levels. We used the 5-mm depth tomographic settings available on the current Heidelberg device. The focus point was set at the top of the intraretinal vascular bundle at the top of the optic disk. The diffusible nature of fluorescein made localization difficult. Thus, we assessed only ICG signal in later (≥5hr) experiments.

### Analysis of mean ONH diameter, rNAION induction severity and maximum edema

**Fig 1:**
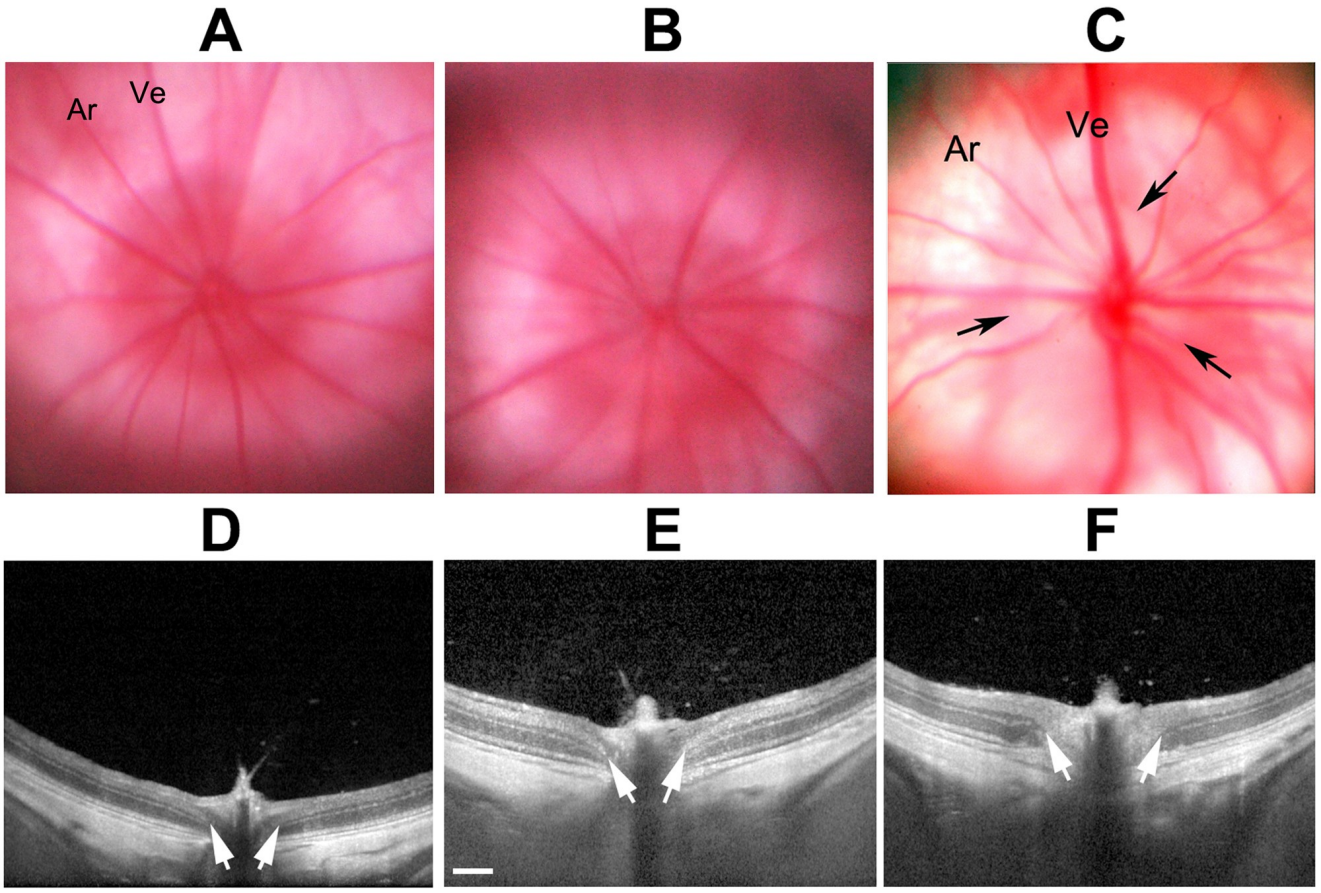
SD-OCT detectable changes following rNAION. **A-C**: Sequential *en face* views of the rodent retina and intraocular portion of the ON in a pre-induced naïve (A), a 5hr post-induction (B), and a 2d post-induction animal (C). **A**. Naïve (uninduced). The ONH has a reddish flush, with the ON border is definable as a slightly lighter color against the retina (Ret). Retinal arteries (ar) and veins (ve) are normal in appearance, and are not engorged. **B**. 5hr post-induction. The ONH is slightly lighter in appearance but still flat against the retina. Retinal veins are slightly engorged. **C**. 2d post-induction. The ON is edematous and pale. The border of the intraocular ON is poorly defined. **D-F**: Sequential cross-section individual slice views of the same nerves shown above, using the Heidelberg instrument and a commercially available rodent fundus contact lens. **D**. Naïve. The intraocular ONH diameter is determined by the INL-INL cross-sectional distance (arrows). **E**. 5hr post-induction. The ONH diameter is slightly enlarged (arrows), and there is engorgement of the vascular tip above the ONH. **F**. 2d post-induction. The ONH diameter is significantly expanded in the intraocular region (arrow), and the nerve fiber bundle has become lighter (edema) and the layers less distinct.

Two days post-induction, animals were re-examined following contact lens application at the slit-lamp and by the Heidelberg device using the IR-OCT display. Using the plano-convex contact lens, both retinal *en face* and cross-sectional imaging can be performed. We used a 15-degree scan through the central retina and ONH, and a 25-slice analysis, with 30 scans averaged/slice (Fig 1D–1F).

This approach enabled analysis of mean naïve ONH diameter, peripapillary nerve fiber layer thickness, and expansion of the ONH diameter using the instrument-included calipers. The mean ONH diameter was determined by averaging three sequential cross-sections of the ONH with the greatest distance separating both sides of the inner nuclear layer (Fig 1D–1F; arrows).

### Retinal ganglion cell stereology

Thirty days post induction, animals were euthanized, and eyes and ONs isolated and post-fixed in 4% paraformaldehyde (PF)-PBS (pH 7.4). PF-PBS-fixed retinas were isolated, and RGCs were immunostained using a primary goat polyclonal anti-Brn3a antibody (Santa Cruz Biotechnology, Dallas, TX), followed by fluorescent labeling with a donkey anti-goat Cy3 secondary antibody (Jackson Immunoresearch, West Grove, PA), flat-mounted and stereologically counted as previously described, using a Nikon Eclipse E800 fluorescent microscope (Nikon, Melville NY) with motorized stage, driven by a stereological imaging package (StereoInvestigator, Ver 10.0; Microbrightfield Bioscience, Williston, VT). Stereological analysis was performed using the Stereo Investigator 10 package, with counts in each eye greater than that required by the Schmitz-Hof equation for statistical validity [21].

## Results

### Systemic parameters in induced animals

Induced animals had a mean rectal temperature of 37.2±0.3° C (range 36.9–37.5° C; n = 11). Two animals died after induction (n = 2), one of which had a rectal temperature of 35° C and the other, 36.1° C (both considerably below the mean). Mean Hct was 45.6%, with a range from 43.5 to 51%. The outlier animal with an Hct of 51% had a rectal temperature of 36.9° C and had a minimal (if any) rNAION induction, as measured by SD-OCT mean diameter of 457.3μm and 3.5% RGC loss 30d post-induction. A Pearson correlation was performed with pre-induction Hct and correlating with ultimate RGC loss. This yielded an r^2^ value of 0.240, which was insignificant. Thus, neither temperature nor Hct consistently correlated with relative induction severity.

### SD-OCT imaging and visual examination at 5 hours post-induction cannot quantify ultimate rNAION lesion severity

We imaged both naïve- and 10-second-induced eyes at both 5hr and 2d postinduction, comparing both the *en face* and cross-sectional appearances of the visualizable ONH using both color fundus photography and SD-OCT. Results are shown in Fig 1.

The naïve eye shows a narrow ONH with a reddish color (Fig 1A, ON). The retinal veins (Ve) are narrow, and the arteries are about ½ the diameter of the veins.

Five hours post induction, little color change is apparent in the color fundus photo of the ONH in the *en face* view (compare 1A, naïve, with 1B, 5hr); the amount of ON edema by 5hr is minor, with almost no expansion of the interneural diameter (compare distance between arrows in naïve 1D with those at 5hr post induction, 1E), but with mild blurring of the ONH in cross-section compared with naïve ONH. Two days post induction (Fig 1C, *en face*, and 1F, cross-section), the ONH is visibly edematous (Fig 1C (2d); compare with 1A (naïve)). This correlates with expansion of the intraneural diameter (compare 1D [naïve], with 1F (2d post induction)). Thus, although SD-OCT can detect both subtle early (5hr) and later (2d) edema-related changes associated with rNAION, the early imaging cannot be used to determine ultimate severity.

We also analyzed progressive visual changes in ON disk appearance in animals over a 1-week period by slit-lamp biomicroscopy and correlated these changes with ultimate (30d) RGC loss, to identify the minimum time required for development of maximal ONH edema. Because of the stress of daily general anesthesia required for this analysis, the animals used in this experiment were limited to a small sighting group (n = 3), and we used only inhaled anesthesia for the few minutes required to keep the animal stable for visual analysis (although not for SD-OCT). We found that although optic disk edema was easily detectable by three trained observers by 1d post-induction, 2 days post-induction was the earliest time point that all observers independently could verify optic disk edema in all animals, with edema resolving by day 7 post-induction. Table 1 shows the results of this study.

**Table 1:** Visual characteristics of the intraocular portion of the rat ON following rNAION induction.

Rat (SD)	Induction Time	IVC?	1 hr post-induction	3 hr post-induction	5 hr post-induction	1 day post	2 day post	5 day post	7 day post	% RGC loss
1	10s	Yes	No changes	Slight disk pallor	Pale disk, Swollen Vs	2+ disk edema/ swollen Vs	**3+ Disk edema**	3+ disk edema	1+	53.2
2	10s	Yes	Slight disk pallor	No changes	Pale disk, Swollen Vs	3+ disk edema/ swollen Vs	**3+ disk edema**	3+ disk edema	1+	76.4
3	10s	Yes	No changes	Slight disk pallor	No disk changes	1–2+ disk edema/ swollen Vs	**3+ disk edema**	3+ disk edema	1+	73.9

Sprague-Dawley rats (SD) were induced for 10 seconds, and the intraocular portion of the ON was evaluated independently by three graders from 1hr to 1 week post-induction using a slit-lamp biomicroscope. ONH edema was apparent at 1 day and was maximal by 2 days post-induction (*2 day post; indicated in bold). RGC loss at 30d was moderate-severe in all three animals. IVC: initial vein constriction at the optic disk immediately post-induction. Vs: vessels

### rNAION induction reveals an induction threshold effect

The mean ONH diameter of the contralateral (uninduced; naïve) eye was 310±38.2μm (sd), with a range of 254–399μm (n = 29 animals). In contrast, the mean ONH diameter of all 2d post-induced animals induced with the commercially available contact lens (9–11 sec; n = 33 animals) measured by SD-OCT was 511±77.2μm, with a range of 359–665μm. We compared both individual induced-ONH OCT data with subsequent RGC loss data from these eyes, as well as naïve ONH diameter vs RGC loss data from the same animals.

**Fig 2:**
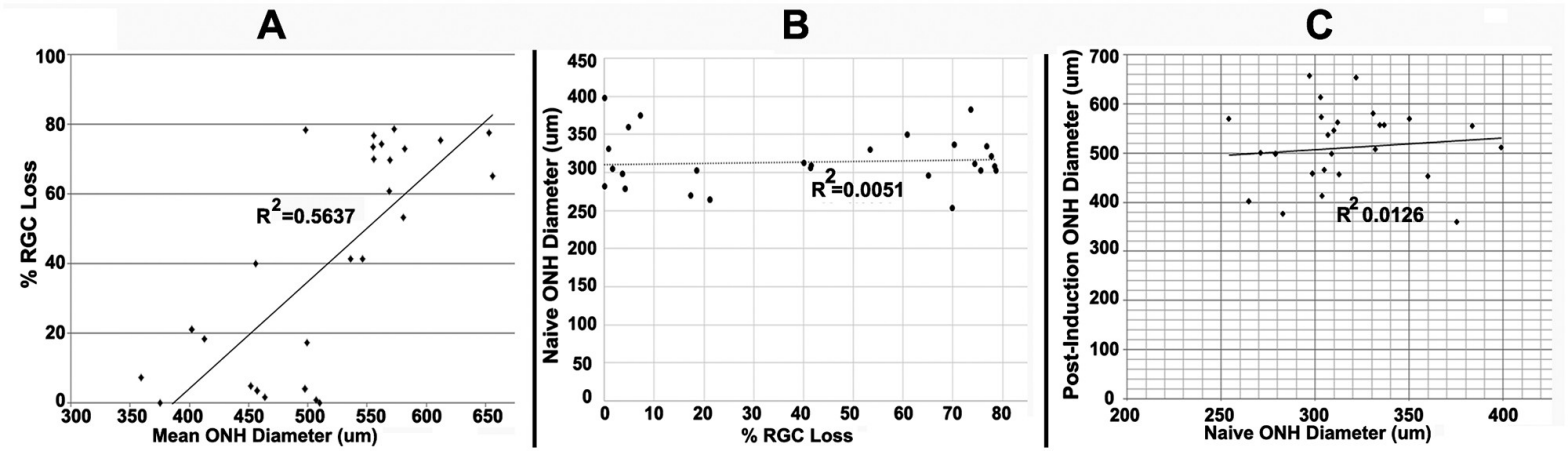
Mean post-induction ONH diameter correlates with RGC loss. A. Correlation of mean post-induction ONH diameter with RGC loss. Post-induction RGC loss is minimal in animals with mean post-ONH diameters <500μm (10/13 animals had RGC loss <20%), whereas relative RGC loss is much greater in induced eyes with mean ONH diameter>500μm; r^2^ is 0.56. **B**. No correlation of pre-induction ONH diameter with subsequent RGC loss; r^2^ is 0.00. **C**. No correlation of ONH diameter between naïve eye and 2d post-induced eye. The r^2^ value is 0.01.

Results are seen in Fig 2.

Analyses were subjected to linear regression, and an r^2^ value was derived for each analysis. Comparing individual post-induction ONH edema levels with relative RGC loss revealed a moderate correlation (r^2^ = 0.5637) of edema severity with RGC loss (Fig 2A). Animals with a mean INL-INL diameter <500μm (189.6μm greater than mean naïve diameter of 310.4μm) had a mean RGC loss of 16.1±5.44% standard error of the mean (SEM) (range, 0–78.3%, n = 14 animals; 13 animals with RGC loss <25%). In contrast, eyes with a mean INL-INL diameter >500μm had a mean RGC loss of 56.2±5.6% (SEM) (range 0–78.6%; n = 19 animals; two animals with RGC loss <25%). This sharp delineation is consistent with a threshold phenomenon associated with a compartment syndrome. In contrast, correlating naïve eye ONH diameter with the percentage of RGC loss in the induced contralateral eye reveals no association between ONH diameter prior to induction and the potential degree of RGC loss following induction (Fig 2B; r^2^ = 0.0051). Unlike clinical NAION, pre-induction (naïve) ONH diameter does not appear to contribute significantly to the degree of severity of the compartment syndrome generated 2d post induction in the rNAION model (Fig 2C; r^2^ = 0.0126).

### rNAION lesion severity shows a steep response of RGC loss with laser induction time

RGC stereology using Brn3a(+) staining of retinal flat mounts of naïve eyes revealed a mean count of 1539.004±36.3 (SEM) Brn3a(+)RGCs/unit area with a calculated mean total number of 77,101±4748 standard deviation (sd) RGCs, and a range of 66,800–85,733 (n = 53 eyes). Mean inter-eye variation (left-right comparison) was ~2.5%. There can be as much as 8% (sd) variability among naïve-eye retinal counts from animals in the same groups.

**Fig 3:**
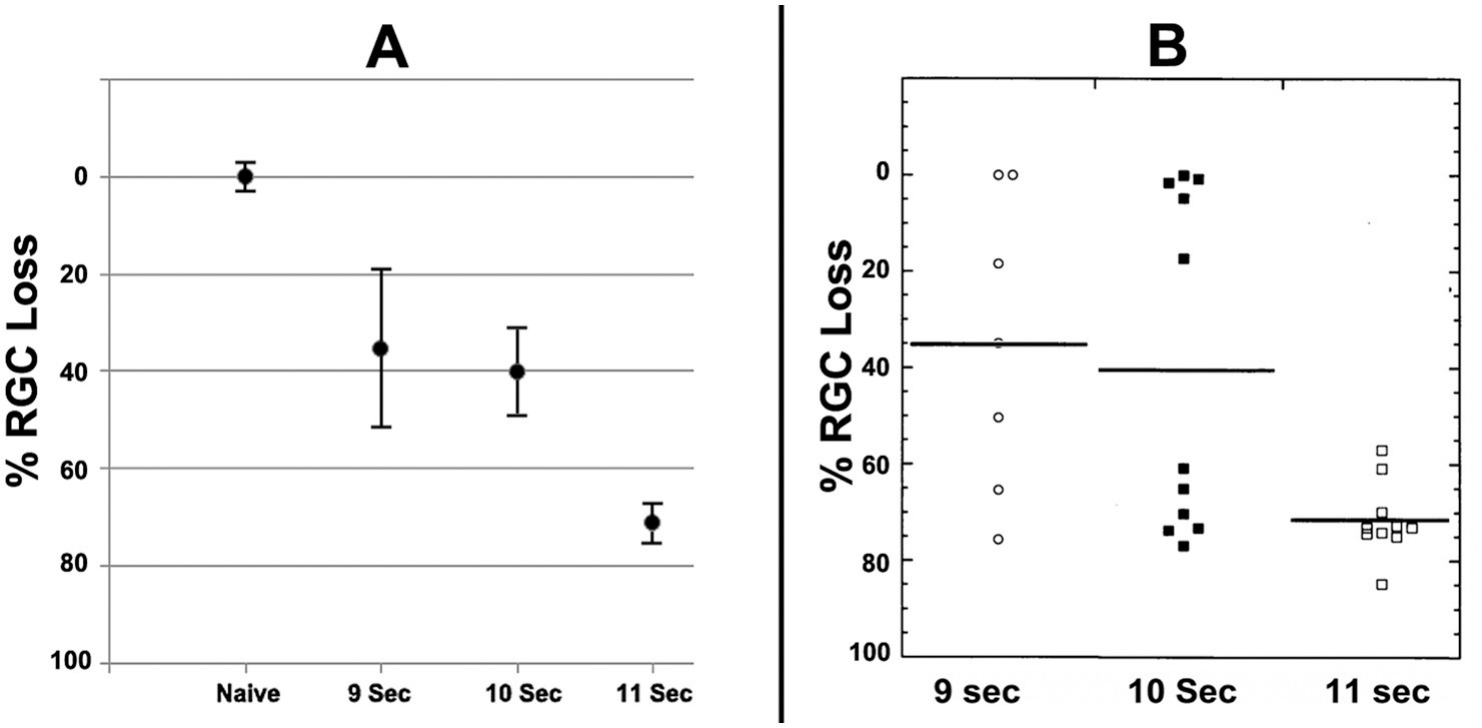
rNAION induction severity in response to laser induction time. **A**. Comparison of RGC counts in animals induced for 9, 10 and 11 seconds. The X-axis gives the condition, whereas the Y axis gives the % remaining RGCs. Naïve RGC numbers are given as 100±2.5% (SEM). There is a progressive loss of RGCs with increasing laser induction time from 9–11 seconds (Fig 3A). **B**. Scatterplots of individual retinal counts for each eye. There is considerable variability in RGC loss with induction times <11 seconds, and a threshold effect (RGC loss) is clearly discernable in animals treated for 9 or 10 seconds, with ~half of all animals exhibiting significant RGC loss (defined as <20%).

We compared RGC loss with induction times ranging from 6 to 11 seconds (Fig 3). Six-second induction times yielded no appreciable RGC loss (rNAION induced 99.2±3.6% (SEM) compared with mean RGC value in uninduced contralateral control eyes; n = 3). Eleven-second rNAION induction times generated the most consistent ONH lesions, resulting in a mean 71.42 ± 2.53% (SEM) RGC loss (Fig 3B; black bar on the far right). An induction time of 10 seconds generated ONH lesions with a mean loss of 40.32±7.79% (SEM), and 9 seconds of induction generated a loss of 34.50±17.89 (SEM). Thus, reduced induction times are associated with decreased RGC loss; however, there is a steep relationship of average RGC loss response to rNAION induction time.

A scatterplot of the data shown in Fig 3B is far more revealing than a simple bar graph. These data reveal that as the relative induction severity (laser induction time) fell below 11 seconds, a significant number of animals in each group failed to show any RGC loss (RGC remaining), with the number of animals without any RGC loss increasing as induction times decreased. For example, nearly all animals induced for 11 seconds had significant RGC loss (Fig 3B; RGC loss), but 3/11 (27.3%) animals induced with laser for 10 seconds failed to show any RGC loss (defined as ≤3% difference from the contralateral eye). An even higher 42.8% of animals induced for 9 seconds showed no or only minimal RGC loss, suggesting an rNAION threshold induction effect. We have previously noted that animals induced for 11 seconds or longer occasionally can develop branch or central retinal vein occlusions, resulting in retinal ischemia and associated RGC loss [22], but these complications were not seen in the current study, possibly related to the use of the commercially available fundus contact lens described above.

#### Early biomarker nalysis using IVFA and ICGA to identify relative post-rNAION lesion severity

**Fig 4:**
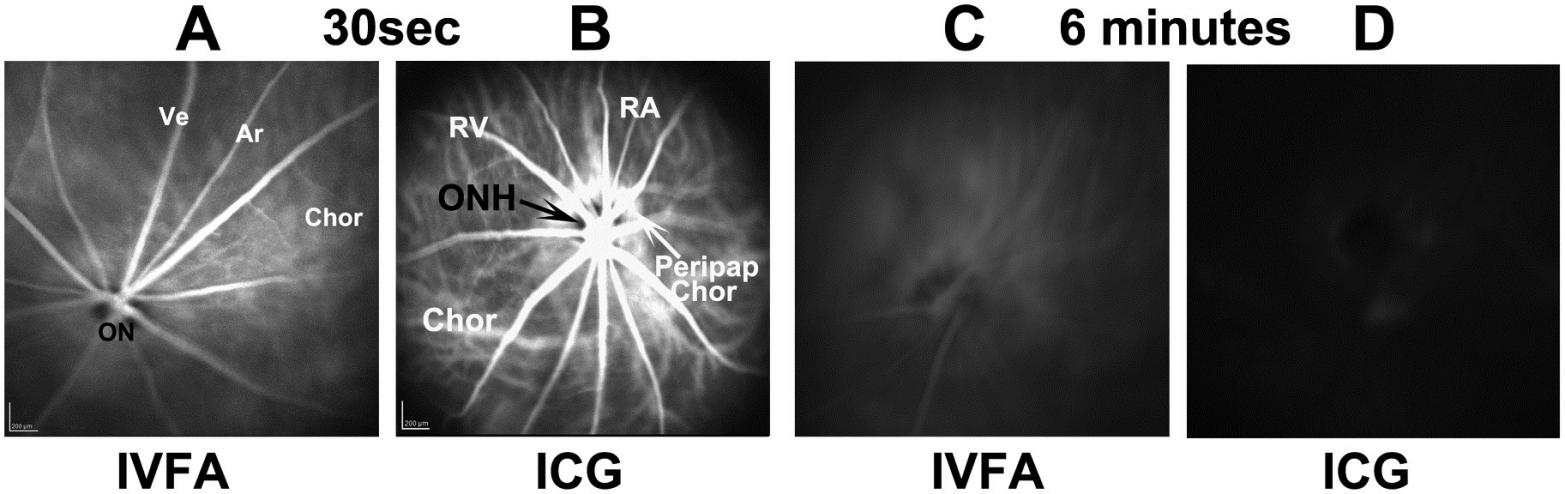
Information obtained through fluorescein and ICG angiography of uninduced (A-D) retinae and ONH. All imaging was done at similar times with the same fundus lens. A and B: IVFA and ICGA imaging, 30 sec post-injection. The IVFA reveals inner retinal vasculature (Ar and Ve). The intraocular optic nerve head (ON) appears as a dark center in the IVFA panel. B. ICG of uninduced eye. The dark ON head (ONH; arrow) is surrounded by a ring of peripapillary choroidal (Peripap Chor) fluorescence surrounding the rat ONH. C and D: IVFA and ICGA photos, respectively, of the same animal, taken 6min post injection. The ON and veins (Ve) are barely visible by IVFA, whereas little if any fluorescence remains in the ICGA channel.

Fluorescein is largely unbound to serum proteins and rapidly diffuses through tissue if vascular barriers are disrupted, whereas ICG accumulates near the areas of vascular leakage, enabling identification of different features of vascular decompensation. Following IVFA in naïve (uninduced) eyes, fluorescein signal is constrained within the retinal and choroidal vasculature (Ve and Ar) at 30 seconds post-injection (Fig 4A).

There is poor resolution of the choroidal signal (Fig 4A, Chor). The ONH is dark and remains so in naïve eyes even after several minutes, revealing that fluorescein leakage does not occur in the uninduced ONH. Fluorescein signal is greatly reduced by 6 minutes post-injection (Fig 4C). The ICG signal 30 seconds post IV administration (Fig 4B) reveals a more complex vascular perfusion pattern in both superficial retinal and choroidal vasculature. The choroidal vascular pattern includes additional strong signal in the peripapillary region. Similar to fluorescein, ICG signal rapidly declines after injection; naïve eyes have minimal ONH or retinal signal 6 minutes post injection. Thus, data obtained from ICGA provide more complete vascular information, enabling a more complete analysis.

**Fig 5:**
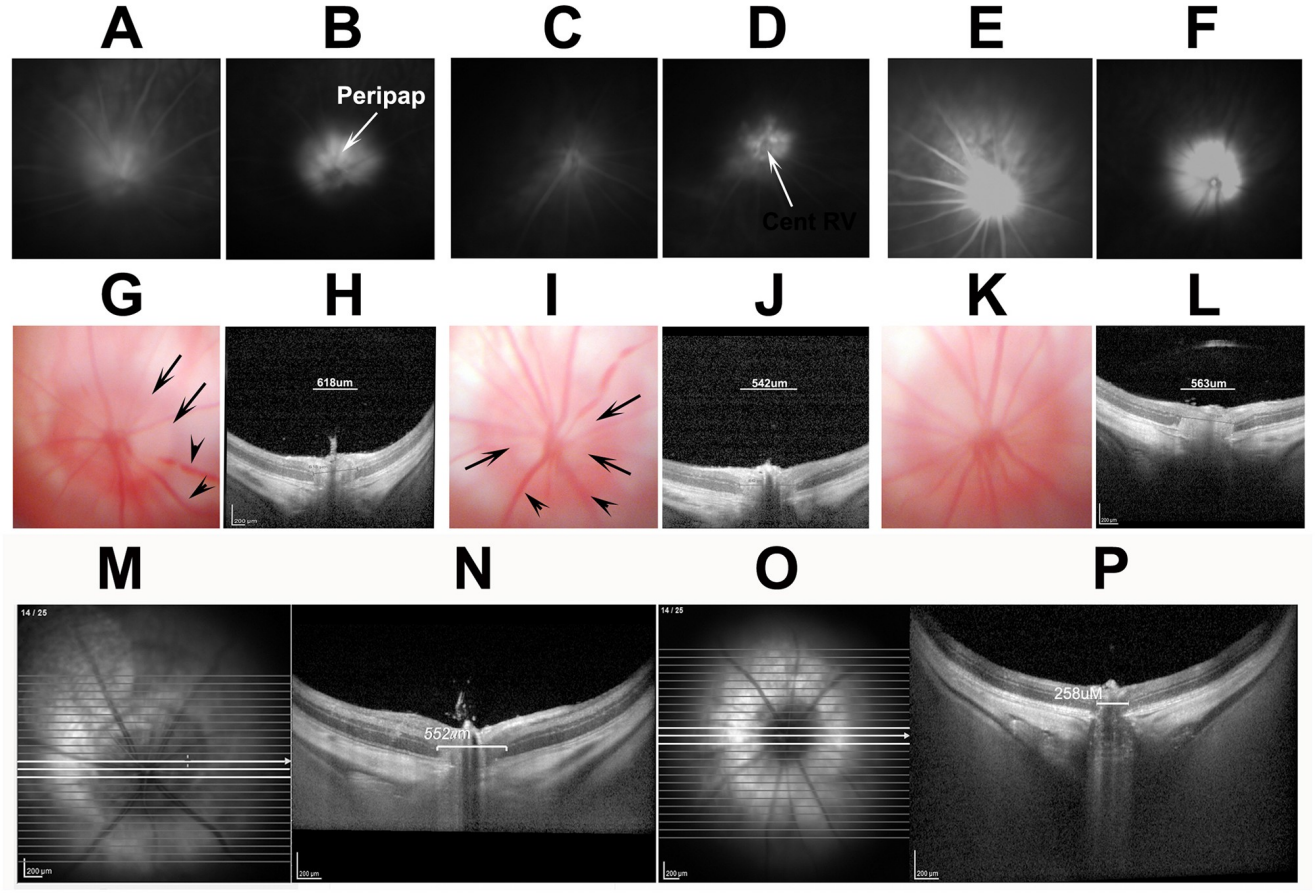
Comparison of characteristics of three similarly induced (9 second) rNAION animals. All animals evaluated at the same time with the same lens and parameters. A-F: paired IVFA (A,C,E) and ICGA (B,D,F), respectively, of three induced eyes 5hr post-induction (ICG 5mg/ml/kg). Diffuse fluorescent signal is present following fluorescein administration above the disk in all three animals, whereas the ICGA photos reveal localized ICG leakage pattern limited to the ONH, the central retinal vascular bundle, and the peripapillary retinal pigment epithelium (RPE). The inner retinal vasculature is apparent in one animal by fluorescein (E; compare with A and C). G-L: Two day post-induction color fundus photo- (G,I,K) and SD-OCT-based cross-sectional image- (H,J,L) pairs taken at the ON centers from the same eyes seen in A-F. There is apparent ON edema in all three eyes (G,I,K) and demonstrable, but varied amounts of expansion of the ON cross-sectional diameter of all three eyes (H,J,L; individual quantification bars seen above the ON). There also is dilation of some retinal veins (arrowheads, G and I) and blurring of the vasculature (see arrows in G and I). There was 46.3%, 60.1% and 28.3% RGC loss at 30 days in these three animals by stereology. M-P: examples of 2d post-induction edema quantification. Following a 150-segment SD-OCT scan using a fundus contact lens, the INL-INL distance from the three middle cross-sections with the greatest distances are averaged. M and N: imaging from a 9 second eye. O and P: Imaging from the contralateral uninduced eye. Scale bars in M-P: 200μm.

We evaluated characteristics of rNAION-induced (n = 9) eyes for vascular leakage at 5 hours (Fig 5A–5F), and ONH edema and fundus appearance at 2 days post induction (Fig 5G–5L), comparing IVFA and ICGA results for each individual animal (shown in pairs: 9-second inductions; A-B; C-D and E-F).

**Fig 6:**
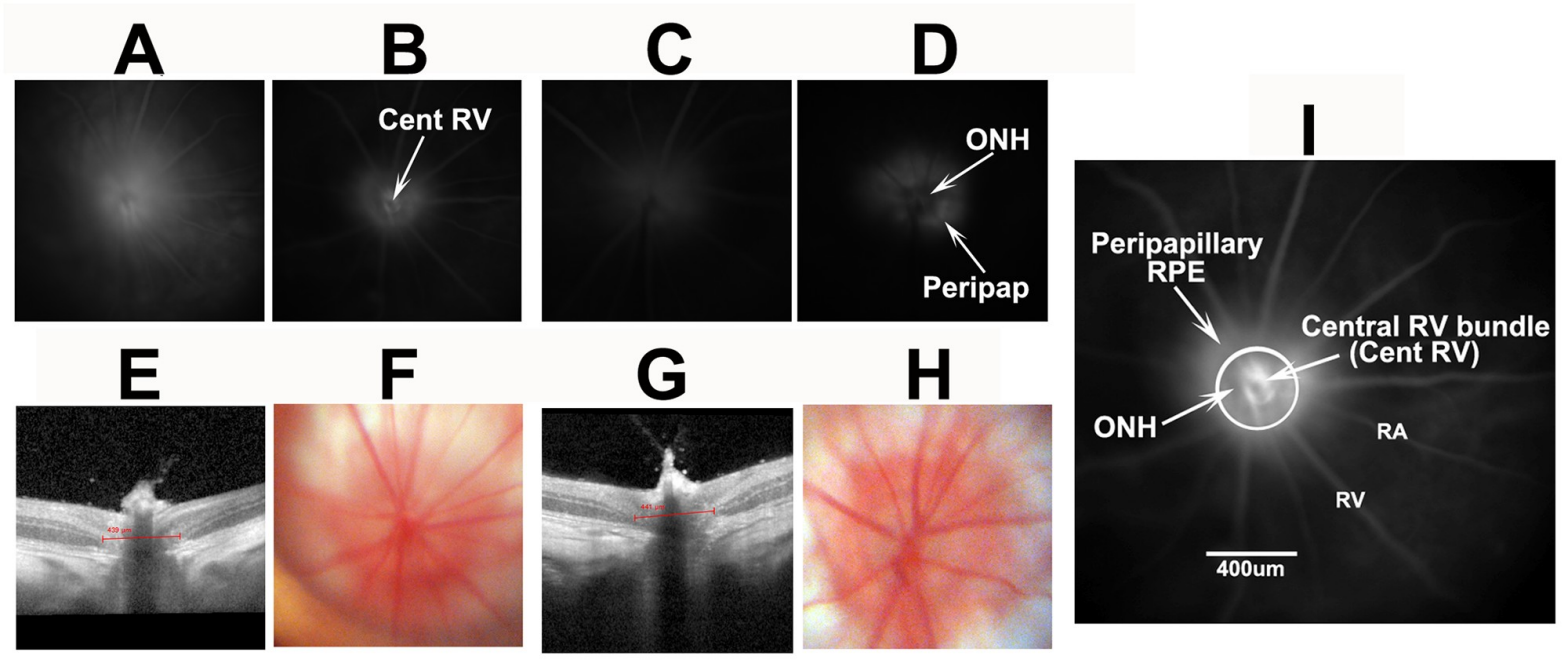
Analysis of two 6-second rNAION-induced animals (no RGC loss) at 2 days post-induction. A-D: 5hr leakage analysis of fluorescein (A and C) and ICG (B and D). Moderate diffuse fluorescein leakage is seen in one animal (A) but almost none in the other animal (C), whereas ICG signal at the same time post-injection reveals a more complex leakage pattern, with dye localization around the ONH. E and G: SD-OCT cross-sectional photos. F and H: Fundus color photos. ONH edema is apparent by quantification of the INL-INL distance (439μm and 441μm, respectively, for F and H). There is mild blurring of the ONH margin in the fundus photo F, and none in H. I. Higher magnification of panel 5D, with increased contrast and signal showing ICG leakage. The circle indicates roughly 500um. There is mild signal at the central vascular bundle (site I) in the center of the ONH. The ONH (site II) itself is relatively dark against the surrounding area, revealing reduced leakage relative to the peripapillary RPE (peripapillary sub-RPE) (site III). Scale bar in I: 400μm.

We compared 5hr fluorescence patterns with the degree of SD-OCT-based ON edema measures 2d post induction and RGC loss at 30d. This enabled us to determine if relatively early dye leakage correlated with lesion severity and RGC loss and to compare the relative ease of interpretation for the two dyes. All eyes induced for 9 seconds showed ONH leakage at 5 hours, >500μm diameter of ONH edema at 2d, as measured by increase in the mean INL-INL gap (618μm, 542μm, and 563μm respectively) (panels 5H,J,L) and variable but significant RGC loss (28.3, 60.1 and 28.3%), respectively. The 9-second-induced eyes with the lowest fluorescent signal (Fig 5C) yielded more RGC loss (60.1%) than similarly induced eyes with more leakage signal (compare 5A and E; RGC loss 46.3 and 28.3%, respectively), An eye induced for 6 seconds (Fig 6A) with a leakage signal similar to that of an eye induced for 9 seconds had no RGC loss at 30 days (compare Fig 5A, 9 sec, with Fig 6A. 6 sec). Thus, the amount of fluorescein leakage has little correlation with the degree of RGC loss.

We also evaluated the relative degree of vasculature leakage associated with milder induction (6 seconds) (Fig 6), all of which had no 30d RGC loss. There was less ICG leakage signal at 5 hours (Fig 6B and 6D), compared with 9-second-induced animals (compare 6B and 6D, 6 seconds with Fig 5B and 5D, 9 seconds), but vascular leakage at 6 seconds also was highly variable. Six-second-induced eyes also showed less ONH edema at 2 days, with ONH expansion below 500μm (439μ and 441μm, respectively) and minimal visible fundus changes. Fluorescein dye leakage was relatively nonspecific, with poor resolution of the sites of leakage.

A closer evaluation of ICG dye leakage from the optic disk 5 hours post-rNAION induction revealed a complex leakage pattern response at 6 minutes post-dye administration in both 9- and 6-second inductions (compare Figs 5B, 5D, 5F, 6B and 6D).

The leakage pattern is best illustrated by the enlarged ONH-ICG photo in Fig 6I. The strongest leakage signal was generated by peripapillary choroidal vasculature surrounding the ONH under the retinal pigment epithelium (RPE), but there also was ICG leakage from the ONH, compared with its surround (5D; ONH, arrow). Fluorescent signal also was present at the center of the ONH by the central retinal vascular bundle (Fig 6I, Cent RV). Thus, the rNAION induction model generates a complex vascular decompensation response with contributions from the three components: the central vessels emerging from the ONH, the ONH itself, and the choroidal tissue surrounding the ONH, which normally is obscured by the RPE.

In contrast to 9-second inductions, 6-second-induced eyes 2d post induction had gross minimal changes in the ONH and surrounding peripapillary retina by slit-lamp visualization at 2 days (Fig 6F and 6H), with minimal ONH edema (defined as <500μm, 439μm and 441μm, respectively; see panels 5E and 5G) and no discernable RGC loss. Naïve (uninduced) eyes had a mean ONH INL-INL gap width of 327.5μm. We also used ICG-coupled ONH OCTA, which can be used to image tissue planes at defined depths above and below a particular focal fixation point. Tomography with a 5mm range focused on the anterior posterior pole tissue did not allow better dissection of the leakage at different depths. Thus, ICGA did not enable appreciably better early prediction of ultimate RGC loss than did FA, despite providing additional information about leakage sites.

## Discussion

Similar to human NAION, the rNAION model expresses significant variability in its severity and expression in different individuals. This occurs despite maximum consistency of the rNAION induction technique, including optimizing thermal support, using a commercially generated lens, limiting animals to a single sex, normalizing the injected dye volume, maintaining a consistent time from IV dye injection to laser induction, and optimization of laser focus by direct observation. We found no evidence of an association between lesion severity and Hct, core temperature, heart rate, or respiratory rate during induction.

The finding that RGC loss in the rNAION model is associated with a threshold expansion of the ONH is consistent with the ‘compartment syndrome’ theory of the pathophysiology of clinical NAION; ie, there is a threshold level of ONH edema that results in compression of the ONH capillaries in both human NAION and rNAION. However, assuming that both ONHs of each animal are similar in diameter, the rNAION model, unlike human NAION, shows no correlation of preinduction ONH diameter with subsequent ONH edema (r^2^ = 0.0126), and subsequent RGC loss (r^2^ = 0.0051), suggesting that, unlike the small cup-to-disk ratio that is the ‘disk at risk’ that predisposes humans to NAION, the rodent model does not possess a homologous association. Our current data suggest that although the rNAION model is similar to human NAION in terms of the development of ONH edema, RGC loss, and other pathophysiological processes such as inflammation [23], the pre-induction diameter of the rodent ONH contributes little if anything to the ultimate development of the pathology. It also should be noted that the animals used in these studies are outbred Sprague Dawley rats. Although this potentially could increase variability in lesion severity, our small group study using inbred (Wistar) rats, also showed no improvement in lesion consistency with respect to measured ONH edema, even when eyes of the same animal were induced sequentially (mean Wistar ONH edema 11-sec induction = 595.8±31.3μm right eye v. 609.6±90.4μm left eye (sd); n = 6 animals). There also were differences in residual RGC numbers between rNAION-sequentially induced (1 week apart) eyes of the same animals using the same parameters (74.07±10.58% (sd) RGC loss first eye v. 36.24±38.56% (sd) RGC loss contralateral eye). Thus, there must be other variables that alter the severity of rNAION that have yet to be determined. The ability of the SD-OCT device to quantify rodent ONH diameter, although precise when comparing between eyes within a species, may be less accurate than that for humans, as the Heidelberg device optics and software are designed for human analysis, despite excellent visualization with the current setup. However, comparing SD-OCT images with histological sections of an even a smaller rodent species (mouse) has yielded a correlation of 0.9042 [24], suggesting that Heidelberg imaging of rat eyes coupled to the contact lens is likely to be at least as good as, and likely even better results than in mouse. Comparing eyes within the same species, the relative degree of error is likely to be consistent, and, thus, the ONH measurements obtained from one animal can be compared with animals of the same species, age, and sex.

Dye leakage studies revealed ONH leakage at 5 hrs post-induction, but these also were not informative with respect to the degree of either lesion severity or ultimate RGC loss. ICG signal enabled considerably more information than did IVFA, which largely generated a cloud of dye in front of the ONH. The ICG pattern in uninduced eyes reveals a dense peripapillary choroidal vascularization pattern (Fig 4B), consistent with previous corrosion casting studies demonstrating a major arteriolar supply to the peripapillary choroid that perfuses the ONH [25].

ICG angiography revealed that the vascular-associated leakage that results in the ultimate rNAION lesion is correspondingly complex, with three vascular sites contributing to overall ONH edema: 1) capillaries and larger vessels in the overlying retina; 2) deeper intra-ON capillaries partially obscured by the overlying large retinal veins and arteries (see Fig 4B); and 3) the dense vascular complex within the peripapillary choroid (see Fig 4B). There also was a contribution by the peripapillary RPE, which may be directly affected by laser energy. Disappointingly, the degree of early peripapillary and intraneural dye leakage was not associated with the amount of ultimate ONH edema or RGC loss. This may be due to additional variables yet to be discovered or controlled, such as subtle differences in the angle of laser exposure to the ONH; the insensitivity of current methods to identify the most relevant site of vascular decompensation for progression despite depth sectioning approaches such as OCT and OCTA; or, due to the small size of the rodent ONH, variations in ONH capillary density in individual rodent ONHs that contribute to induction sensitivity. This last possibility seems most likely to us, because mice, with smaller ONHs, have an even greater degree of induction variability [26], whereas there is much less induction variability among eyes in Old World primates, which have considerably larger ONHs and more exuberant capillary beds [27]. Based on this increased morphological size, ICGA leakage analysis may prove useful in identifying regional affected areas in humans who present early in the course of NAION.

Animals in previous studies were evaluated for relative lesion severity at 1–2 days post induction using available methods, including direct visualization and SD-OCT *en face* and cross-sectional analyses. ONH cross-sectional analysis of post-induction ONH edema moderately correlates with ultimate RGC loss (r^2^ = 0.56). Selecting animals with mean INL-INL expansion >500μm 2d post-induction eliminates the vast majority of induced animals with RGC loss <10% (see Figs 1 and 2). Our findings suggest that, as in the cases of human NAION and nonhuman primate NAION (pNAION) [7], rNAION-induced RGC loss likely results from edema causing a compartment syndrome at the ONH and the minimum required induction parameters exhibit a threshold effect. Our studies of pNAION showed a significant 1:1 correlation between maximum edema and eventual atrophy, consistent with damage resulting from a compartment syndrome [28]. The ONH diameter-expansion biomarker is likely to prove most applicable in minimizing animal numbers for randomized studies in which treatments begin at least 1–2 days post induction. The majority of studies in this category are neuroregenerative or neuroinflammatory reduction protocols, in which reagents are administered days or even weeks post induction [29]. This approach also may be useful for evaluating early post-induction drug treatment effects such as edema reduction, with sufficient animals, as overall edema is associated with rNAION induction. However, this tool is less likely to be useful in early treatment administration studies, which comprise the majority of current neuroprotective reagents.

In summary, the rNAION model, although useful, must be used with a number of caveats. The model is appropriate in early analyses for identifying edema-reducing therapies, but caution must be taken to include a sufficient number of animals to generate robust data, and induction conditions must be structured to either minimize the number of animals that fail to reach threshold edema levels or include sufficient subjects to overcome this problem. For treatments that can be administered later in the course of the model (ie, neuroregenerative or apoptosis inhibition), ONH edema measurements 1-2d post induction can be used to eliminate non-threshold animals, thus enabling randomization of animals with similar lesion severity into ultimate treatment groups and to reduce the number of study animals that must be carried and processed to final analysis. Other functional approaches, such as OCTA, may prove to be useful in the future for identifying early changes associated with ultimate lesion severity and RGC loss.

## References

[1] Miller NR, Arnold AC. Current concepts in the diagnosis, pathogenesis and management of nonarteritic anterior ischaemic optic neuropathy. Eye. 2014;29(1):65–79. 10.1038/eye.2014.144 24993324 PMC4289822

[2] Oto S, Yilmaz G, Cakmakci S, Aydin P. Indocyanine green and fluorescein angiography in nonarteritic anterior ischemic optic neuropathy. Retina. 2002;22:187–191. 10.1097/00006982-200204000-00009 11927852

[3] Janaky M, Fulop Z, Palffy A, Benedek K, Benedek G. Electrophysiological findings in patients with nonarteritic anterior ischemic optic neuropathy. Clin Neurophysiol. 2006;117:1158–1166. 10.1016/j.clinph.2006.01.013 16551511

[4] Kerr NM, Chew SS, Danesh-Meyer HV. Non-arteritic anterior ischaemic optic neuropathy: a review and update. J Clin Neurosci. 2009;16:994–1000. 10.1016/j.jocn.2009.04.002 19596112

[5] Bernstein SL, Guo Y, Kelman SE, Flower RW, Johnson MA. Functional and cellular responses in a novel rodent model of anterior ischemic optic neuropathy. Invest Ophthalmol Vis Sci. 2003;44:4153–62. 10.1167/iovs.03-0274 14507856

[6] Goldenberg-Cohen N, Guo Y, Margolis FL, Miller NR, Cohen Y, Bernstein SL. Oligodendrocyte Dysfunction Following Induction of Experimental Anterior Optic Nerve Ischemia. Invest Ophthalmol Vis Sci. 2005;46: 2716–2725. 10.1167/iovs.04-0547 16043843

[7] Chen CS, Johnson MA, Flower RA, Slater BJ, Miller NR, Bernstein SL. A Primate Model of Nonarteritic Anterior Ischemic Optic Neuropathy (pNAION). Invest Ophthalmol Vis Sci. 2008;49: 2985–2992. 10.1167/iovs.07-1651 18326695 PMC2754050

[8] Bernstein SL, Johnson MA, Miller NR. Nonarteritic anterior ischemic optic neuropathy (NAION) and its experimental models. Prog Retin Eye Res. 2011;30:167–187. 10.1016/j.preteyeres.2011.02.003 21376134 PMC3367439

[9] Tesser RA, Niendorf ER, Levin LA. The morphology of an infarct in nonarteritic anterior ischemic optic neuropathy. Ophthalmology. 2003;110:2031–2035. 10.1016/S0161-6420(03)00804-2 14522783

[10] Pangratz-Fuehrer S, Kaur K, Ousman SS, Steinman L, Liao YJ. Functional rescue of experimental ischemic optic neuropathy with alpha B-crystallin. Eye (Lond). 2011;25:809–817. 21475310 10.1038/eye.2011.42 PMC3178147

[11] Huang T-L, Wen Y-T, Chang C-H, Chang S-W, Lin K-H, Tsai R-K. Efficacy of Intravitreal Injections of Triamcinolone Acetonide in a Rodent Model of Nonarteritic Anterior Ischemic Optic NeuropathyTriamcinolone in Rat Ischemic Optic Neuropathy. Invest Ophthalmol Vis Sci. 2016;57:1878–1884. 10.1167/iovs.15-19023 27082303

[12] Kapupara K, Huang T-L, Wen Y-T, Huang S-P, Tsai R-K. Optic nerve head width and retinal nerve fiber layer changes are proper indexes for validating the successful induction of experimental anterior ischemic optic neuropathy. Exp Eye Res. 2019;181:105–111. 10.1016/j.exer.2018.12.007 30557569

[13] Prieto R, Carceller F, Roda JM, Avendano C. The intraluminal thread model revisited: rat strain differences in local cerebral blood flow. Neurol Res. 2005;27:47–52. 10.1179/016164105X18214 15829158

[14] Shmonin A, Melnikova E, Galagudza M, Vlasov T. Characteristics of cerebral ischemia in major rat stroke models of middle cerebral artery ligation through craniectomy. Int J Stroke. 2014;9:793–801. 10.1111/j.1747-4949.2012.00947.x 23205677

[15] Nicholson JD, Puche AC, Guo Y, Weinreich D, Slater BJ, Bernstein SL. PGJ2 Provides Prolonged CNS Stroke Protection by Reducing White Matter Edema. PLoS One. 2012; 10.1371/journal.pone.0050021 23284631 PMC3527449

[16] Greene SA, Thurmon JC. Xylazine—a review of its pharmacology and use in veterinary medicine. J Vet Pharmacol Therap. 1988;11:295–313. 10.1111/j.1365-2885.1988.tb00189.x 3062194

[17] Lee HB, Blaufox MD. Blood Volume in the Rat. J Nuc Med. 1985;26:72–76. 3965655

[18] Ianacone DC, Felberg NT, Federman JL. Tritiated Fluorescein Binding to Normal Human Plasma Proteins. JAMA Ophthalmol. 1980;98:1643–1655. 10.1001/archopht.1980.01020040495022 7425929

[19] Muci-Mendoza R, Arevalo JF, Ramella M, Fuenmayor-Rivera D, Karam E, Cardenas PL, et al. Optociliary veins in optic nerve sheath meningioma. Indocyanine green videoangiography findings. Ophthalmology. 1999;106:311–318. 10.1016/S0161-6420(99)90055-6 9951483

[20] Yoneya S, Saito T, Komatsu Y, Koyama I, Takahashi K, Duvoll-Young J. Binding properties of indocyanine green in human blood. Invest Ophthalmol Vis Sci. 1998;39:1286–1290. 9620093

[21] Schmitz C, Hof PR. Design-based stereology in neuroscience. Neuroscience. 2005;130:813–831. 10.1016/j.neuroscience.2004.08.050 15652981

[22] Hirabayashi K, Tanaka M, Imai A, Toriyama Y, Iesato Y, Sakurai T, et al. Development of a Novel Model of Central Retinal Vascular Occlusion and the Therapeutic Potential of the Adrenomedullin-Receptor Activity-Modifying Protein 2 System. Am J Pathol. 2019;189:449–466. 10.1016/j.ajpath.2018.10.021 30658846

[23] Salgado C, Vilson F, Miller NR, Bernstein SL. Cellular inflammation in nonarteritic anterior ischemic optic neuropathy and its primate model. Arch Ophthalmol. 2011;129:1583–1591. 10.1001/archophthalmol.2011.351 22159678 PMC3494739

[24] Knott EJ, Sheets KG, Zhou Y, Gordon WC, Bazan NG. Spatial correlation of mouse photoreceptor-RPE thickness between SD-OCT and histology. Exp Eye Res. 2011;92:155–160. 10.1016/j.exer.2010.10.009 21035444 PMC3169798

[25] Morrison JC, Johnson EC, Cepurna WO, Funk RH. Microvasculature of the rat optic nerve head. Invest Ophthalmol Vis Sci. 1999;40:1702–1709. 10393039

[26] Bernstein SL, Guo Y. Changes in cholinergic amacrine cells after rodent anterior ischemic optic neuropathy (rAION). Invest Ophthalmol Vis Sci. 2011;52:904–910. 10.1167/iovs.10-5247 20847114 PMC3053113

[27] Olver JM, Spalton DJ, McCartney AC. Microvascular study of the retrolaminar optic nerve in man: the possible significance in anterior ischaemic optic neuropathy. Eye. 1990;4:7–24. 10.1038/eye.1990.3 2323480

[28] Johnson MA, Miller NR, Nolan T, Bernstein SL. Peripapillary Retinal Nerve Fiber Layer Swelling Predicts Peripapillary Atrophy in a Primate Model of Nonarteritic Anterior Ischemic Optic Neuropathy. Invest Ophthalmol Vis Sci. 2016;57:527–532. 10.1167/iovs.15-17880 26868755 PMC4758301

[29] Sozmen EG, Rosenzweig S, Llorente IL, DiTullio DJ, Machnicki M, Vinters HV, et al. Nogo receptor blockade overcomes remyelination failure after white matter stroke and stimulates functional recovery in aged mice. Proc Natl Acad Sci *USA*. 2016;113:E8453. 10.1073/pnas.1615322113 27956620 PMC5206535

